# Evaluation of curcumin-loaded chitosan nanoparticles for wound healing activity

**DOI:** 10.5599/admet.1897

**Published:** 2023-08-25

**Authors:** Smita Kumbhar, Rupali Khairate, Manish Bhatia, Prafulla Choudhari, Vinod Gaikwad

**Affiliations:** 1 Department of Pharmaceutical Analysis, DSTS Mandal’s College of Pharmacy, Solapur, India; 2 Department of Pharmaceutical Chemistry, Bharati Vidyapeeth College of Pharmacy, Kolhapur, India; 3 Department of Pharmaceutics, National Institute of Pharmaceutical Education and Research (NIPER), Hajipur, India

**Keywords:** Skin regeneration, turmeric, ionotropic gelation, chitosan, nanoparticles, topical administration

## Abstract

**Background and purpose:**

Wound healing is a biological process that can be difficult to manage clinically. In skin wound healing, the interaction of many cells, growth factors, and cytokines reveals an outstanding biological function mechanism. Wound healing that occurs naturally restores tissue integrity, however, it is usually restricted to wound repair. Curcumin synthesised in a chitosan matrix can be used to heal skin sores.

**Experimental approach:**

The ionotropic gelation procedure required crosslinking chitosan with a tripolyphosphate (TPP) crosslinker to generate curcumin nanoparticles encapsulated in chitosan.

**Key results:**

The nanoparticles were between 200 and 400 nm in size, with a strong positive surface charge and good entrapment efficacy, according to SEM and TEM investigations. Curcumin and chitosan compatibility was investigated using FTIR spectroscopy. All batches showed consistent drug release, with the F5 batch having the highest curcumin release, at 75% after 16 hours. On L929 cells, scratch assays were utilised to assess wound healing. Wound closure with widths of 59 and 65 mm with curcumin and 45 and 78 mm with curcumin-loaded chitosan nanoparticles was seen after 24 and 48 hours of examination.

**Conclusions:**

According to the findings, prepared curcumin chitosan nanoparticles are beneficial in healing skin damage.

## Introduction

The skin is the largest organ and the most formidable barrier to external elements entering the body. Tumor excision, accidents, diabetic ulcers, incisions, thermal, chemical, or electric burns can all cause skin damage. Acute or chronic wounds exist. The fundamental issue with chronic wounds is that they get colonized with germs, which slows or prevents healing. This might be due to a lack of peripheral artery supply, venous drainage problems, or diabetes mellitus [1]. Untreated skin injuries can raise the risk of infection, disability, and even death. Hemostasis, inflammation, proliferation, and remodeling are the four processes involved in wound healing [2-4]. Wound management necessitates regular dressing changes, which patients may resist. Wound management necessitates regular dressing changes, which patients may resist. As a result, wound management requires a proper medication delivery system that can deliver the drug to the desired location and in a regulated manner. As a result, a variety of polymers are now being used to create nanoparticles for innovative drug delivery systems.

Curcumin is a phytopolyphenol known as diferuloylmethane and is the main biological active element in the Curcuma longa plant. Anti-inflammatory, anti-diabetic, anticancer, anti-mutagenic, and wound-healing activities are all present [5-8]. Curcumin's wound-healing benefits are due to its antioxidant, antibacterial, and anti-inflammatory characteristics. It impacts the granulation, collagen deposition, remodeling, and wound closure stages of the wound healing process [9,10]. In human gingival fibroblast cells, it was also demonstrated to repair wounds by boosting transforming growth factor-1 and vascular endothelial growth factors. The most significant obstacles to using this drug therapeutically are its hydrophobic nature, limited bioavailability, and rapid metabolism. Curcumin integrating with chitosan nanoparticles was explored to boost water solubility to overcome these constraints [11].

Chitosan is a hydrophilic polymer derived from the deacetylation of chitin, with repeating units of D-glucosamine and N-acetylglucosamine. Chitosan nanoparticles are widely employed in biomedical applications such as drug administration, vaccine delivery, antibacterial agent delivery, and wound healing [12]. Because of its beneficial qualities, such as non-toxicity, biodegradability, and increased collagen deposition, it has been employed in wound repair [13]. Curcumin with chitosan nanoparticles has a lot of potential for wound healing and skin tissue regeneration [14]. Curcumin nanoparticles have received a lot of interest for their anticancer and wound-healing properties due to their low toxicity and other pharmacological properties, as well as the great attributes that make them a good wound-healing ingredient [15-17].

A lot of research is going on chitosan nanoparticles due to their beneficial effects. Chitosan nanoparticles have been used to deliver hyaluronic acid for targeted therapy for small-cell lung cancer and to make oil-in-water (O/W) hypaphorine nanoparticles for chronic wound healing [18-20]. Curcumin-loaded dextrin sulphate chitosan nanoparticles have also been created to promote curcumin's anticancer action [21]. Curcumin has also been created for wound healing applications in chitosan/pluronic-based membranes [22]. In other studies, chitosan coated on curcumin-loaded PLGA (poly lactic-co-glycolic acid) nanoparticles increased apoptosis and cytotoxicity much more than free curcumin. In combination with *Pongamia pinnata*-mediated silver nanoparticles in chitosan nanoparticles, curcumin has anti-inflammatory properties and can combat infections in wound treatment [23].

In this study, an improved method for synthesizing curcumin-loaded chitosan nanoparticles was established, and the in vitro wound healing efficiency on L929 fibroblast cells was studied.

## Experimental

### Materials

Sigma-Aldrich Chemicals, Bangalore, provided Chitosan (low molecular weight, 75 % deacetylation, 1 % viscous solution), Curcumin, and Tripolyphosphate. National Centre for Cell Science, Pune (NCCS), Maharashtra, provided L929 fibroblast cell lines. Biological Scientific Solutions, New Delhi, supplied Dulbecco's phosphate buffer saline. All other compounds used were analytical grade and came from domestic suppliers.

### Formulation of curcumin-loaded chitosan nanoparticles

Curcumin-loaded chitosan nanoparticles were created using the ionotropic gelation process based on the electrostatic interaction between positively and negatively charged molecules using TPP (Tripolyphosphate) as a crosslinking agent. The following steps were used to make curcumin-loaded chitosan nanoparticles. The following steps were used to make curcumin-loaded chitosan nanoparticles.

In deionized water, 5 ml of 0.2 % w/v TPP solution was prepared. Curcumin was dissolved in methanol to get 0.1 % w/v concentration, which was then premixed with 0.05 % w/v chitosan dissolved in 2 % v/v glacial acetic acid at pH 5 with continuous stirring for 30 minutes. The TPP solution was then dropped into the aforementioned solution and stirred at 1000 rpm for 1 hour at room temperature. The final product was centrifuged for 1 hour at 10,000 rpm at 20 °C. The nanoparticles were resuspended in distilled water and dried in a freeze dryer at -48°C until complete drying with cryoprotectant ethylene glycol to obtain pellets. The formulated nanoparticles were used for further evaluation tests.

### Characterization of curcumin-loaded chitosan nanoparticles

#### Scanning electron microscopy

The size of nanoparticles was examined by scanning electron microscopy (SEM) (Hitachi 4000 Plus, Japan). Particles were suspended on a glass slide and kept under vacuum and scanning electron microscopy was operated at 10KV accelerated voltage.

#### Transmission electron microscopy

Transmission electron microscopy (TEM; JEM-2000 EX; JEOL, Japan) was used to identify the shape and surface morphology of the particles. Images were obtained with a digital camera, and the diameter of individual nanoparticles was measured to quantify particle size.

### Zeta potential

The zeta potential of nanoparticles is a key metric for determining their stability for nanoparticles [24]. It was determined using the electrophoretic light scattering method (Horiba SZ-100, Japan) at 25.3 °C with a voltage of 3.3 V and a conductivity of 0.603 mS/cm.

### Spectroscopic characteristics

Infrared light is absorbed by molecules in Fourier transform infrared spectroscopy (FTIR). The FTIR spectrum indicates the total molecular makeup [25]. Fourier transform infrared spectroscopy used a mercury cadmium telluride detector to record the characteristic peaks of functional groups of curcumin, chitosan, and curcumin-loaded chitosan nanoparticles (Brucker Alpha 2, Germany). The spectra of samples were dispersed in KBr and the discs were made and FTIR spectra were recorded in the range of 400 to 4000 cm^-1^.

### Thermal characteristics

Thermal analysis is a method of determining physical qualities as a function of temperature to determine the physical and chemical properties of polymers, geological materials, and other materials [26].

Differential scanning calorimetry was used to investigate the thermal behaviour of curcumin-loaded chitosan nanoparticles (Perkin Elmer 4000, Germany). By heating 3-4 mg of samples at a rate of 20 to 420 °C/min, the transition temperature and enthalpies of the samples were determined. The typical thermal peaks were obtained.

### Encapsulation efficiency of curcumin-loaded chitosan nanoparticles

The encapsulation efficiency of fabricated nanoparticles was determined by UV spectrophotometer. The amount of drug in the suspension was analysed by centrifuging nanoparticles at 1000 rpm for 30 minutes and measuring the concentration of the drug in the supernatant at 425 nm. The concentration of the drug was analysed after necessary dilutions. The encapsulation efficiency (EE), the amount of medicine successfully captured inside a micelle or nanoparticle, is known as the encapsulation efficiency, computed using Equation (1).


(1)

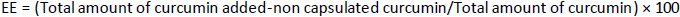



### Curcumin release profile from nanoparticles

The Franz diffusion cell was used to measure curcumin release from nanoparticles in phosphate buffer saline pH 7.4 at various time intervals. The Franz diffusion cell's receiving compartment was filled with phosphate buffer saline. The donor and receiving compartments were separated by a Nylon 6,6 membrane (0.2 μm pore size). The drug release study was carried out for 16 hours. 20 mg of curcumin-loaded chitosan nanoparticles were weighed and placed on the membrane. After 0, 1, 3, 5, 8, and 16 hours, the specified volume of sample solution was extracted and replaced with phosphate buffer saline in the same volume. The amount of curcumin emitted from nanoparticles was measured using a UV/Visible spectrophotometer at 425 nm.

### In vitro wound healing by scratch assay method

In-vitro cell migration on L929 cells was studied by scratch assay method [27] to assess the wound healing capacities of curcumin and curcumin-loaded chitosan nanoparticles. Cells were seeded onto 6 well plates at a density of 2×10^5^ cells per well and cultivated overnight at 37 °C. The cells were rinsed in Dulbecco's phosphate buffer saline (DBPS), used as culture media and scratched with a sterile 200 μl tip. L929 murine fibroblast cells were given 5 μg/ml standard cipladine, 100 μg/ml curcumin, and 100 μg/ml nanoparticles. Photographs taken with an inverted microscope and a digital camera revealed cell movement and morphological alterations. The experiments were carried out in threes (n ¼ 3). SAGLO software was used to examine the width of the scratch and the wound, which was closed at various intervals (0, 24, and 48 hours), was measured.

## Results and discussion

### Morphological characteristics

**Figure 2.**
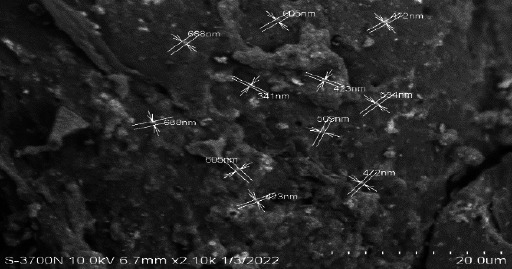
SEM images of curcumin loaded chitosan nanoparticles

**Figure 3.**
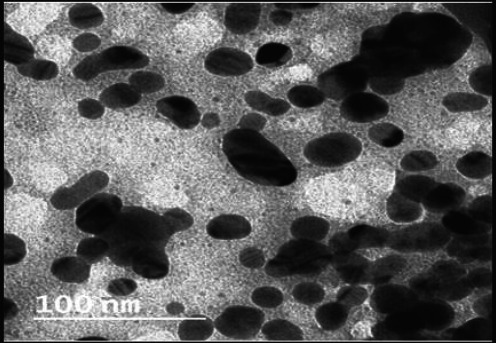
TEM image of curcumin-loaded chitosan nanoparticles

Figure 2 depicts the findings of the morphological analyses of formed nanoparticles. The size range of produced nanoparticles was 400-600 nm, and most particles were spherical, according to SEM analysis. Particles were smaller than 100 nm and had a smooth surface, according to the TEM analysis (Figure 3).

### Zeta potential

**Figure 4.**
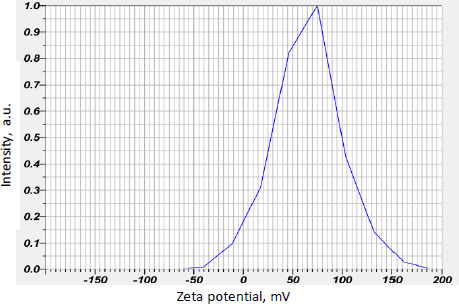
Zeta potential of curcumin-loaded chitosan nanoparticles

Curcumin-loaded chitosan nanoparticles had a mean zeta potential value of 64.9 mV, indicating stability. The mean graph indicating the mean zeta potential value is shown in Figure 4.

### Spectroscopic assessment

**Figure 5.**
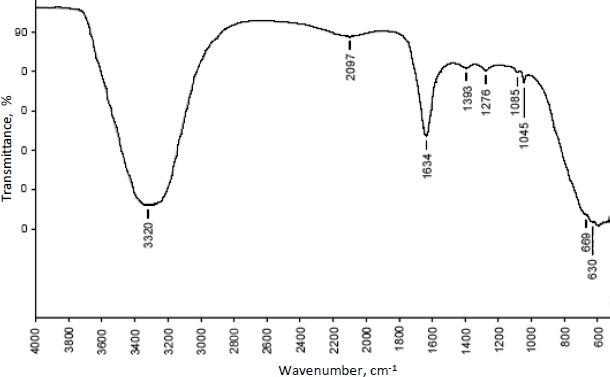
FTIR spectra of curcumin-loaded chitosan nanoparticle

**Figure 6.**
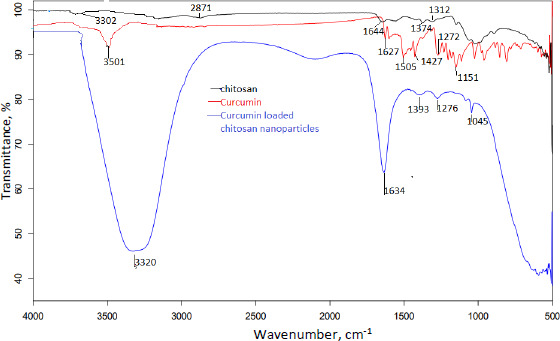
Overlay of FTIR spectra of curcumin, chitosan and formulated nanoparticles

The compatibility of excipients and drugs utilised in nanoparticle formulation was determined using FTIR analysis. Figures 5 and 6 show the FT-IR overlay spectrum of curcumin, chitosan, and curcumin-loaded chitosan nanoparticles.

Curcumin's main peaks were found at 3501 cm^-1^, which was assigned to the free OH group, 1627 cm^-1^, which was attributable to the existence of stretching vibrations of the C=C group, and 1204 and 1272 cm^-1^, which revealed the presence of the C-O-C group. Due to OH/NH stretching vibration, the characterisation peaks of chitosan were found at 3302 cm^-1^, a single and weak band. Weak bands found at 1539-1574 cm^-1^ were caused by the NH_2_ group bending. The existence of the CN functional group is indicated by a peak at 1374 cm^-1^, while the presence of the C-O-C functional group is indicated by medium peaks at 1064 and 1024 cm^-1^.

Different and strong peaks for curcumin-loaded chitosan nanoparticles were observed at 1634 cm^-1^ and 1045 cm^-1^ by comparing the spectra of curcumin and chitosan. There were also shifts in the peak at 3302 to 3320 cm^-1^, 1272 to 1276 cm^-1^, 1204 to 1045 cm^-1^ and 1374 to 1393 cm^-1^.

### Thermal properties

**Figure 7.**
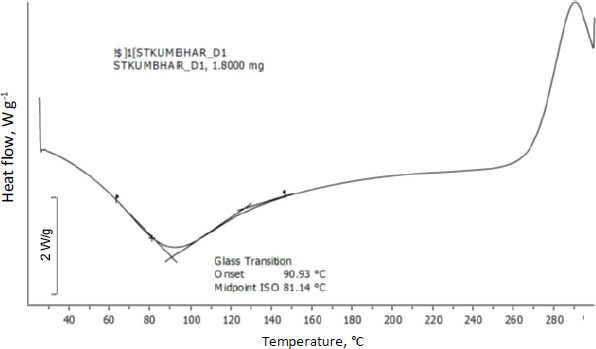
DSC spectra of chitosan

**Figure 8.**
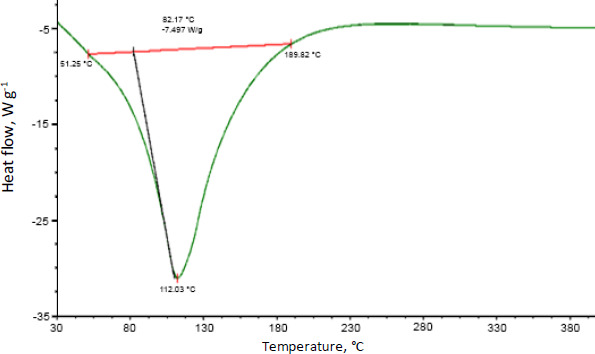
DSC spectra of curcumin-loaded chitosan nanoparticles

Differential scanning calorimetry was used to assess the thermal behaviour of curcumin-loaded chitosan nanoparticles. Glass transition of chitosan was observed at 90 °C. Moreover, an exothermic peak was observed at 289 °C attributed to the degradation of amine units from the chitosan (Figure 7). Figure 8 shows the abrupt endothermic peak at 112.08 °C corresponds to the formed nanoparticles' transition temperature.

### Encapsulation efficiency

The following table shows the encapsulation efficiency of all batches (See Table 1). Compared to all other batches, the F6 and F5 batch has the highest loading efficiency compared to other batches seen in the table (Table 1).

### Drug release profile of curcumin

**Figure 9.**
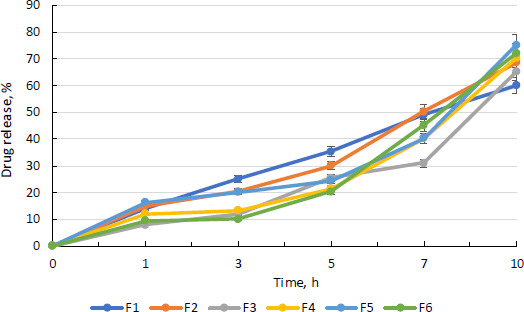
Drug release profile of curcumin-loaded chitosan nanoparticles

Figure 9 depicts the medication release pattern for all batches. For 16 hours, the medication release was monitored. The graph below demonstrates that nanoparticles released curcumin at a steady rate for 10 hours.

According to the diffusion analysis, all batches except the F3 batch release more than 40 % curcumin in 8 h. After 10 hours, the F5 batch has the highest curcumin release, at 75.00 %. The more sustained and consistent release of drug in F5 batch may be due to the more amount of drug that has been entrapped in nanoparticles.

### Wound closure assay by microscopy and image analysis

On L929 fibroblast cells, the width of scratch and in vitro wound closure by migration of cells at the scratch site were investigated for untreated cells, cipladine (standard), curcumin, and curcumin-loaded chitosan nanoparticles. The following photos depict cell migration after 48 hours. When compared to unfabricated curcumin, curcumin loaded in chitosan nanoparticles performed better. The following table shows the results of the scratch assay (Table 2). According to the findings, the scratch treated with nanoparticles had more cells migrating to the wound site and showed greater wound closure than curcumin alone. This could be because of chitosan and curcumin synergistic effect.

**Figure 10.**
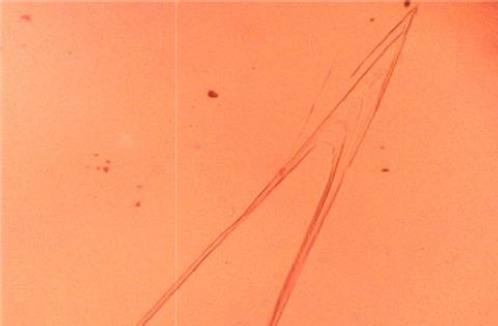
Cell migration analysis of untreated cells

**Figure 11.**
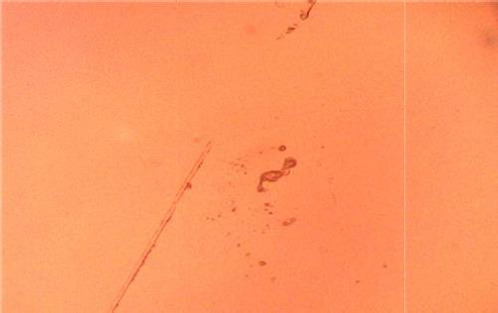
Cell migration analysis of standard cipladine (5 μg/ml)

**Figure 12.**
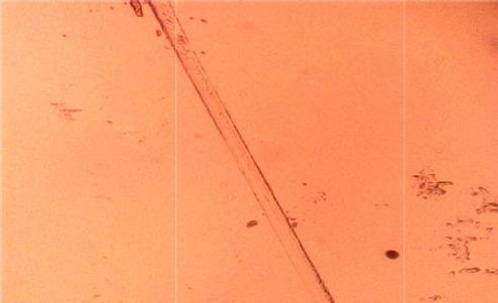
Cell migration analysis of curcumin (100 μg/ml)

**Figure 13.**
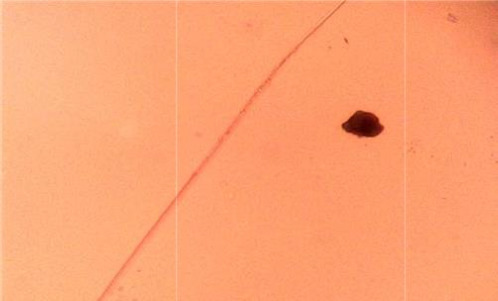
Cell migration analysis of curcumin-loaded chitosan nanoparticles (100 μg/ml)

Cell migration analyses of all the above samples are shown in Figures 10-13 and wound sections treated with control, standard, curcumin and curcumin-loaded chitosan nanoparticles at 0, 24 and 48 h.

## Discussion

The skin is the largest human organ, and drug penetration through it is determined by particle size. Curcumin has been employed in the creation of nanoparticles as a natural, biocompatible, and bioactive substance having antioxidant and antibacterial characteristics. Because of polar lipids, skin is negatively charged. Skin's negative charge attracts positively charged chitosan polymer, allowing drugs and nanoparticles to penetrate deeper into the skin [28]. Curcumin-loaded chitosan nanoparticles were created utilizing an ionotropic gelation technique with TPP as a crosslinker in this study [29].

The particles were nanosized, with a strong positive surface charge, as shown by zeta potential measurements. This guarantees that nanoparticles are stable. The entrapment efficiency of all batches of manufactured nanoparticles was around 80 %. The F5 and F6 batches have shown EE of around 90 %. It shows that the medication is well entrapped in the chitosan polymeric matrix. The F5 batch had the highest drug release, which may be due to the high entrapment of drug in the F5 batch and all batches had a consistent drug release pattern. The prolonged release of curcumin from nanoparticles is expected to aid wound healing while minimizing dressing changes.

The main peaks of curcumin (C=C and OH) were detected using FTIR spectroscopy, indicating that curcumin was loaded in the core of chitosan nanoparticles. The lowered transition temperature of the chitosan moiety in the thermal analysis of curcumin-loaded chitosan suggests that the drug was positioned between the polymeric chains. The scratch assay method was also used to test in vitro wound healing activity. Compared to nonfabricated curcumin, curcumin loaded in chitosan nanoparticles showed better wound closure due to more fibroblast cells migrating at the wound site.

Compared to Mohammad Abdel Hakeem *et al*. [30], our study has the highest encapsulation efficiency and zeta potential. The %encapsulation of the drug was 67 % and +26.66 mV zeta potential in their study and 90.15 % and +64.9 mV in ours. In comparison to Bhunchu S. e*t al*., the EE of formed nanoparticles in our investigation was more than 80 %, whereas in Bhunchu S. et al. work, the EE was less than 50 % and the zeta potential was less than +5 mV [31]. It is greater than 60 mV in our investigation, implying that the nanoparticles created using our approach are more stable. Furthermore, compared to curcumin alone, curcumin-loaded chitosan nanoparticles showed higher cell migration in a scratch experiment of wound closure. Compared to Mohammadmahdi Mobaraki *et al*. [32], our study revealed increased cell migration at 100 g/ml after 48 hours and wound closure at 1 mg of formed nanoparticles in their study.

## Conclusions

The ionotropic gelation process was used to produce curcumin-loaded chitosan nanoparticles successfully. The data of zeta potential suggests good stability of nanoparticles. The DSC results suggest the drug has been physically entrapped and EE of all batches was more than 60 %, indicating good entrapment of drug throughout the polymer. Chitosan and curcumin are compatible according to vibrational spectroscopy, some peaks in the formulation are shifted, suggesting that the polymer and crosslinking agent interacted to form the matrix. The in vitro drug release profile of formulated nanoparticles shows more than 50 % release of curcumin after 10 h and shows a sustained release effect. When formed nanoparticles are compared with curcumin in an in vitro scratch experiment, a greater number of cells have migrated at the wound site and thus, the scratch has closed better compared to the curcumin drug after 48 h.

## Figures and Tables

**Figure 1.**
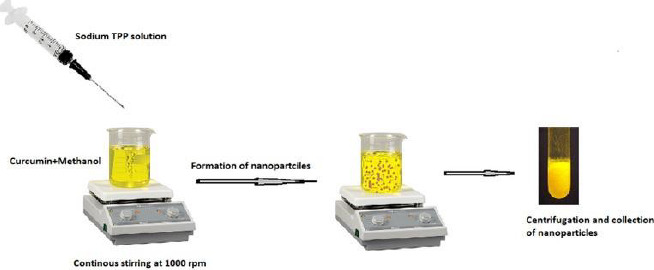
Graphical representation of ionotropic gelation method

**Table 1.** Encapsulation efficiency of formulated nanoparticles

Batches	Content, mg ml^-1^		Encapsulation efficiency, %
	Chitosan	Curcumin	
F1	0.5	0.5	67.80
F2	0.5	1	70.7
F3	0.5	1.5	83.01
F4	0.5	2	86.56
F5	0.5	2.5	90.15
F6	0.5	3	89.62

**Table 2.** Wound closure by scratch assay method.

Name of Sample	Width of wound closed, mm	
	After 24 h	After 48 h
Control/Untreated cells	47 mm	46 mm
Standard (Cipladine 5 μg/ml)	67 mm	87 mm
Curcumin (100 μg/ml)	59 mm	65 mm
Curcumin-loaded chitosan nanoparticles (100 μg/ml)	46 mm	78 mm
